# miR-21 ablation and obeticholic acid ameliorate nonalcoholic steatohepatitis in mice

**DOI:** 10.1038/cddis.2017.172

**Published:** 2017-04-13

**Authors:** Pedro M Rodrigues, Marta B Afonso, André L Simão, Catarina C Carvalho, Alexandre Trindade, António Duarte, Pedro M Borralho, Mariana V Machado, Helena Cortez-Pinto, Cecília MP Rodrigues, Rui E Castro

**Affiliations:** 1 Research Institute for Medicines (iMed.ULisboa), Faculty of Pharmacy, Universidade de Lisboa, Lisbon, Portugal; 2 Reproduction and Development, Interdisciplinary Centre of Research in Animal Health (CIISA), Faculty of Veterinary Medicine, Universidade de Lisboa, Lisbon, Portugal; 3 Gulbenkian Institute of Science, Oeiras, Portugal; 4 Gastrenterology, Hospital Santa Maria, Lisbon, Portugal

## Abstract

microRNAs were recently suggested to contribute to the pathogenesis of nonalcoholic fatty liver disease (NAFLD), a disease lacking specific pharmacological treatments. In that regard, nuclear receptors are arising as key molecular targets for the treatment of nonalcoholic steatohepatitis (NASH). Here we show that, in a typical model of NASH-associated liver damage, microRNA-21 (miR-21) ablation results in a progressive decrease in steatosis, inflammation and lipoapoptosis, with impairment of fibrosis. In a complementary fast food (FF) diet NASH model, mimicking features of the metabolic syndrome, miR-21 levels increase in both liver and muscle, concomitantly with decreased expression of peroxisome proliferator-activated receptor *α* (PPAR*α*), a key miR-21 target. Strikingly, miR-21 knockout mice fed the FF diet supplemented with farnesoid X receptor (FXR) agonist obeticholic acid (OCA) display minimal steatosis, inflammation, oxidative stress and cholesterol accumulation. In addition, lipoprotein metabolism was restored, including decreased fatty acid uptake and polyunsaturation, and liver and muscle insulin sensitivity fully reinstated. Finally, the miR-21/PPAR*α* axis was found amplified in liver and muscle biopsies, and in serum, of NAFLD patients, co-substantiating its role in the development of the metabolic syndrome. By unveiling that miR-21 abrogation, together with FXR activation by OCA, significantly improves whole body metabolic parameters in NASH, our results highlight the therapeutic potential of nuclear receptor multi-targeting therapies for NAFLD.

Nonalcoholic fatty liver disease (NAFLD) represents a pathological condition resulting from high levels of circulating free fatty acids (FFAs), leading to lipid deposition in the hepatocytes, thus triggering steatosis. Steatosis can then progress to nonalcoholic steatohepatitis (NASH), fibrosis, cirrhosis and hepatocellular carcinoma, ultimately culminating in liver failure. NAFLD strongly associates with obesity and the metabolic syndrome, particularly diabetes and whole body insulin resistance.^1, 2^ Nevertheless, NAFLD pathogenesis and progression are not entirely understood, hampering the development of much needed efficient therapies.

microRNAs (miRNAs or miRs) act as potent post-transcriptional regulators of gene expression.^3, 4^ miR-21 is greatly increased in the liver of NASH patients^5^ and in animal models of NASH.^6, 7, 8, 9^ In hepatocytes, miR-21 is induced by unsaturated fatty acids in a mTOR/NF-*κ*B-dependent manner.^10^ It was recently demonstrated that liver miR-21 has an active role in NASH pathogenesis through inhibition of nuclear receptor peroxisome proliferator-activated receptor *α* (PPAR*α*).^6^ Still, whether miR-21 modulates features of the metabolic syndrome, particularly lipid metabolism and insulin resistance in muscle and adipose tissues, remains unknown. Interestingly, PPAR*α* not only controls lipid catabolism and homeostasis, promoting fatty acid oxidation in the liver, but also stimulates glucose capture in the muscle.^11^ As such, regulation of PPAR*α* by miR-21 in the muscle of NAFLD patients may be a relevant event promoting disease development and progression.

Farnesoid X receptor (FXR) is another nuclear receptor having a key role in NAFLD and NASH pathogenesis. FXR induces PPAR*α*, thus promoting fatty acid *β*-oxidation,^12, 13^ and possesses anti-inflammatory properties, mainly by antagonizing NF-*κ*B signaling.^14^ Of note, FXR-deficient mice develop steatosis and hypertriglyceridemia.^15, 16^ Obeticholic acid (OCA) is a potent agonist of FXR, currently approved for the treatment of primary biliary cholangitis (PBC) and in phase 3 clinical trials for the treatment of NASH. Interim results from the NIH-sponsored FLINT clinical trial showed that OCA has significant beneficial effects on NASH-induced liver damage, ameliorating steatosis, inflammation and fibrosis.^17^ However, OCA treatment was associated with greater hepatic insulin resistance and an increase in the frequency and severity of pruritus, the main adverse event reported by patients. OCA-treated patients also displayed an increase in average total cholesterol and LDL, and a decrease in average HDL; a prospective trial to explore the effect of combined OCA and statin therapy on lipid metabolism in NASH patients has been initiated.

In this study, we aimed to evaluate the exact contribution of miR-21 in the pathogenesis and progression of NASH and to evaluate the therapeutic potential of the simultaneous activation of PPAR*α* as a result of miR-21 ablation, and FXR, by administering OCA.

## Results

### MCD diet-fed miR-21 KO mice display reduced steatosis, inflammation, fibrosis and lipoapoptosis

**Figure 1:**
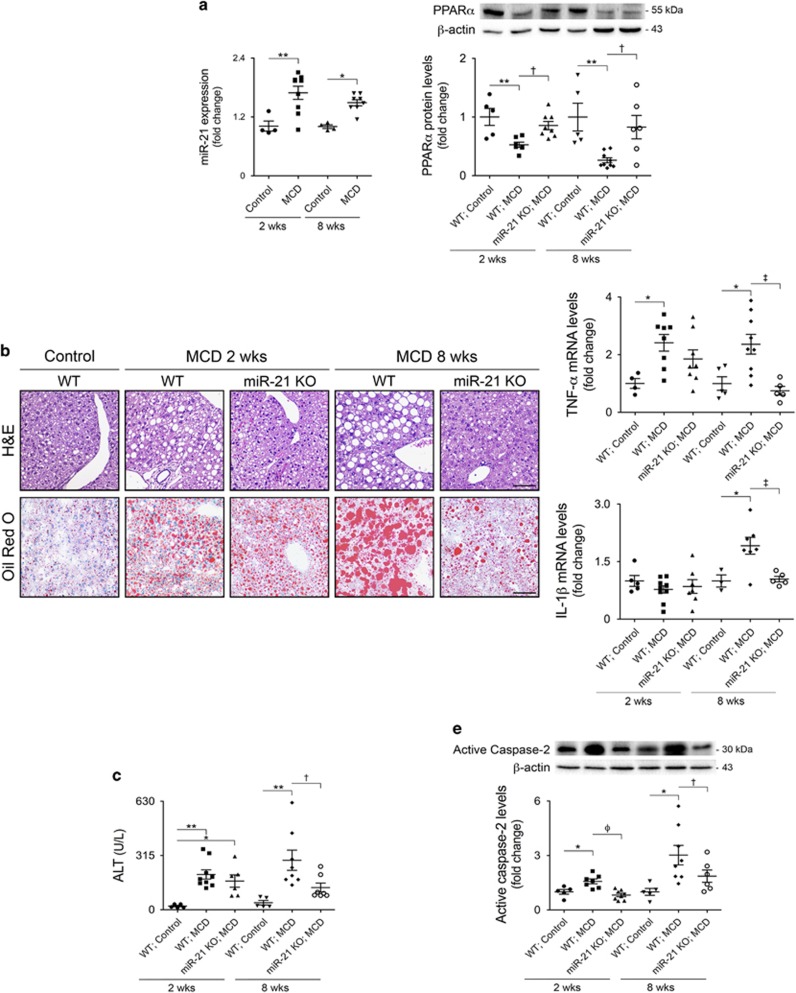

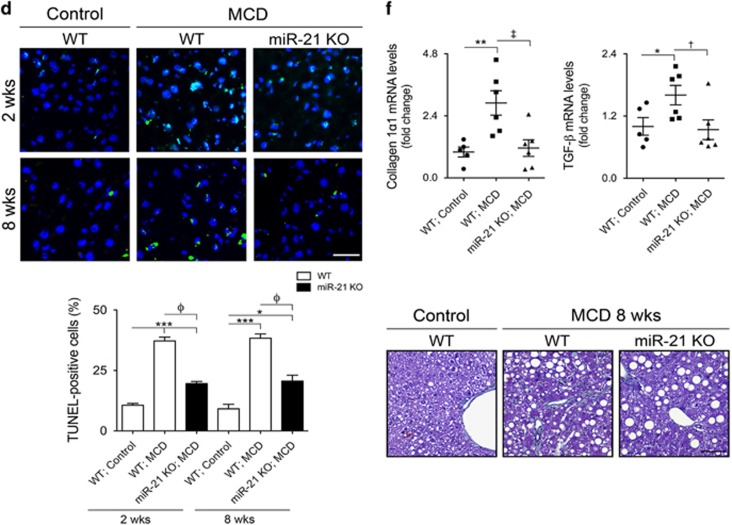
miR-21 ablation protects from MCD-induced steatohepatitis and fibrosis development. C57BL/6 WT and miR-21 KO mice were fed a control (*n*=5 for each time point) or MCD diet (*n*=8–10 for each time point and/or genetic background) for 2 and 8 weeks. (**a**) qRT-PCR analysis of miR-21 (left) and immunoblotting of PPAR*α* (right). Representative blots are shown. Blots were normalized to endogenous *β*-actin. (**b**) H&E and Oil Red-O staining of representative liver sections. Scale bar, 100 *μ*m. (**c**) qRT-PCR analysis of TNF-*α* and IL-1*β*. (**d**) Serum ALT levels. (**e**) TUNEL staining of liver tissue sections. Nuclei were counterstained with *Hoechst* 33258 (blue). Scale bar, 30 *μ*m. Histograms showing the percentage of TUNEL-positive cells (right). (**f**) Immunoblotting of active caspase-2. Representative blots are shown. Blots were normalized to endogenous *β*-actin. (**g**) qRT-PCR analysis of TGF-*β* and collagen-1*α*1 in mouse liver (left) and representative images of Masson's Trichrome stained liver sections from MCD or control diet-fed mice for 8 weeks (right). Scale bar=100 *μ*m. Results are expressed as mean±S.E.M. fold change. *P<0.05, **P<0.01 and ***P<0.001 from control; ^†^*P*<0.05, ^‡^*P*<0.01 and ^*ϕ*^*P*<0.001 compared with respective WT MCD-fed mice

Recent data have shown that miR-21 is increased in the liver of NASH patients and that its inhibition in mice prevents NASH development.^6^ To ascertain the role of miR-21 in NAFLD triggering and progression, wild-type (WT) and miR-21 knockout (KO) mice were fed either a control or methionine-and choline-deficient (MCD) diet for 2 and 8 weeks. miR-21 hepatic levels were significantly increased in WT mice after both 2 and 8 weeks of MCD feeding (*P*<0.05) (Figure 1a left) while remaining undetectable in miR-21 KO mice. Conversely, protein levels of PPAR*α*, a miR-21 direct target contributing to cell injury, inflammation and fibrosis,^6^ progressively decreased in WT MCD-fed mice when compared with WT control-fed mice (*P*<0.05). In addition, MCD diet-fed miR-21 KO mice displayed significantly increased PPAR*α* protein levels in comparison with WT mice, particularly at 8 weeks (*P*<0.05) (Figure 1a right).

After 2 and 8 weeks of feeding, livers from MCD-fed mice displayed typical features of steatohepatitis. At 2 weeks, WT mice already showed mild to moderate steatosis (*P*<0.001; Figure 1b; Table 1). At 8 weeks, WT mice showed an increase in steatosis severity, presenting moderate to severe vacuolation of hepatocytes (*P*<0.001), accumulation of large lipid droplets and pronounced hepatocellular hypertrophy (*P*<0.05). Strikingly, miR-21 KO mice exhibited markedly reduced steatosis, with most animals presenting only mild to moderate hepatocellular vacuolation, with smaller and scattered lipid droplets, as well as a robust decrease in cell hypertrophy (Figure 1b; Table 1).

In WT MCD-fed mice, inflammation and liver injury progressively increased with NASH development, as shown by mRNA levels of TNF-*α* and IL-1*β* (Figure 1c; *P*<0.05) and serum ALT levels (Figure 1d; *P*<0.05). Moderate to severe inflammation was further evidenced by histological analysis (*P*<0.05) (Table 1). These changes were almost abrogated in miR-21 KO mice (*P*<0.01).

FFA-induced hepatocyte apoptosis constitutes a hallmark of NASH.^18, 19, 20, 21^ The percentage of terminal deoxynucleotidyl transferase dUTP nick end labeling (TUNEL)-positive cells in the livers of MCD-fed WT mice increased to ~40% after 2 and 8 weeks (*P*<0.001), in comparison with ~10% in controls, whereas miR-21 KO mice displayed only ~20% of TUNEL-positive cells (Figure 1e). Caspase-2 is markedly upregulated in patients and animal models of NASH, contributing for lipoapoptosis and progression of lipid-induced liver fibrosis.^22, 23^ In agreement, active caspase-2 progressively increased by ~2- and 3-fold in the livers of 2 and 8 weeks MCD-fed WT mice, respectively (*P*<0.05), compared with controls. In contrast, miR-21 KO mice displayed significantly lower levels of active caspase-2 (Figure 1f). Histological analysis also revealed the presence of moderate to severe necrosis in the livers of 8-weeks MCD diet-fed WT mice (*P*<0.05), whereas only a modest increase was observed in miR-21 KO mice (Table 1). In addition, 8 weeks MCD-fed WT mice evidenced deposition of collagen near sinusoids, associated with mononuclear cell infiltration within the liver parenchyma, thus substantiating the development of mild to moderate fibrosis. miR-21 KO mice were protected from fibrosis, as evidenced by decreased mRNA expression of collagen-1*α*1 and TGF-*β* (at least *P*<0.05; Figure 1g, left). Accordingly, deposition of collagen in MCD-fed miR-21 KO mice was minimal and almost all animals were protected against NASH-induced fibrosis (Figure 1g, right; Table 1). Altogether, these results suggest that miR-21 may contribute to NASH development not only through PPAR*α* inhibition, ultimately inducing cell inflammation and fibrosis, but also by promoting liver steatosis and lipoapoptosis.

### miR-21 suppression and FXR activation ameliorates hepatic steatosis and inflammation

Despite efficiently modeling severe NASH with fibrosis,^24^ MCD diet in mice results in weight loss and fails to develop insulin resistance. As such, we next used a complementary model of NASH shown to closely mimic metabolic and histopathological features of human NASH,^25^ constituting a useful model to evaluate the efficacy of prospective new therapies. In this regard, OCA has been successfully investigated in clinical phase II trials with NASH patients (FLINT trial, NCT01265498). Given the pleiotropic spectrum of FXR actions, we hypothesized that a multiple targeting of nuclear receptors may enhance the efficacy and reduce potential side effects of this possible therapeutic approach. WT and miR-21 KO mice were fed either standard (SD) or fast food (FF) diets supplemented with or without OCA for 25 weeks. OCA feeding was shown to activate FXR, as illustrated by the increased mRNA levels of SHP and decreased levels of Cyp7a1 and Srebp-1c (Supplementary Figure S1).

**Figure 2:**
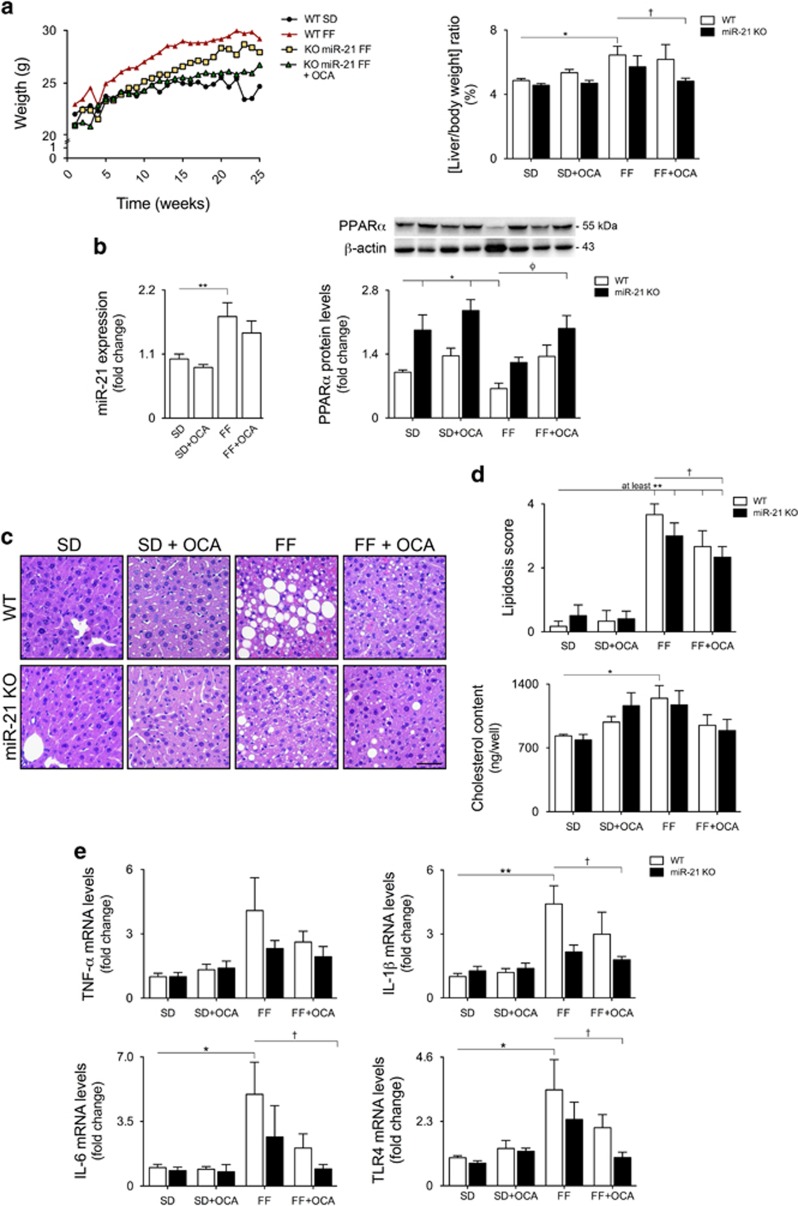
miR-21 ablation and OCA prevent FF-induced hepatomegaly and strongly ameliorate steatosis, cholesterol accumulation and inflammation. C57BL/6 WT and miR-21 KO mice were fed a FF diet (*n*=12) or a control diet (SD; *n*=12), with or without supplementation with OCA (10 mg/kg/day) for 25 weeks. (**a**) Mice body weight (left) and liver to body weight ratio (right). (**b**) qRT-PCR analysis of miR-21 (left) and immunoblotting of PPAR*α* (right). Representative blots are shown. Blots were normalized to endogenous *β*-actin. (**c**) Representative image of H&E-stained liver sections. Scale bar, 100 *μ*m. (**d**) Steatosis score in blinded liver samples (top). Steatosis was graded on a scale 0–4. Liver cholesterol content (bottom). (**e**) qRT-PCR analysis of TNF-*α*, IL-1β, IL-6 and TLR4. Results are expressed as mean±S.E.M. fold change. *P<0.05, **P<0.01 and ***P<0.001 from WT control mice; ^†^*P*<0.05, ^‡^*P*<0.01 and ^*ϕ*^*P*<0.001 compared with WT FF-fed mice. SD; standard diet

After 25 weeks of feeding, WT FF-fed animals gained ~50% of their initial weight (Figure 2a left). miR-21 KO FF-fed mice displayed lower weight increments throughout time, an effect that was even more pronounced on miR-21 KO FF+OCA-fed animals, with body weight increases similar to those of WT mice fed a SD. In addition, WT FF animals developed hepatomegaly, with a significant increase in the liver to total body weight ratio (*P*<0.05 *versus* SD-fed animals). Of note, although miR-21 absence or FXR activation by OCA alone failed to suppress hepatomegaly, miR-21 KO FF+OCA-fed animals displayed liver/body weight ratios similar to SD-fed mice (Figure 2a right). Liver miR-21 expression was significantly upregulated by the FF diet (*P*<0.01), concomitantly with decreased PPAR*α* protein levels (*P*<0.05). Although not significant, OCA slightly decreased miR-21 expression and increased PPAR*α* levels in both diets and genetic backgrounds. PPAR*α* levels were significantly upregulated in miR-21 KO FF+OCA-fed mice in comparison with WT animals (*P*<0.01; Figure 2b).

WT FF-fed animals developed macrovesicular steatosis, presenting an average lipidosis score of 3.67 (*P*<0.001). Although both miR-21 ablation or OCA supplementation resulted in a modest decrease in steatosis, the dual activation of PPAR*α* and FXR resulted in markedly reduced lipid accumulation, diffused microvesicular steatosis and a lipidosis score of 2.33 (*P*<0.05; Figures 2c and d top). In addition, hepatic cholesterol content in WT FF-fed mice increased by ~50% when compared with control animals (*P*<0.05), whereas miR-21 KO FF+OCA-fed mice displayed a robust decrease in cholesterol accumulation within the liver (Figure 2d bottom). Histological analysis further revealed an increase in pro-inflammatory infiltrates within the liver of WT FF-fed mice and a concomitant increase in mRNA levels of pro-inflammatory cytokines TNF-*α*, IL-1*β*, IL-6 and TLR4 (at least *P*<0.05). Although miR-21 abrogation or OCA administration alone reduced expression of these inflammatory markers, this effect was further enhanced in miR-21 KO FF+OCA-fed animals, with cytokine expression reverting to almost control levels (*P*<0.05; Figure 2e).

### Lipid and cholesterol metabolism are positively modulated upon simultaneous activation of PPAR*α* and FXR

**Figure 3:**
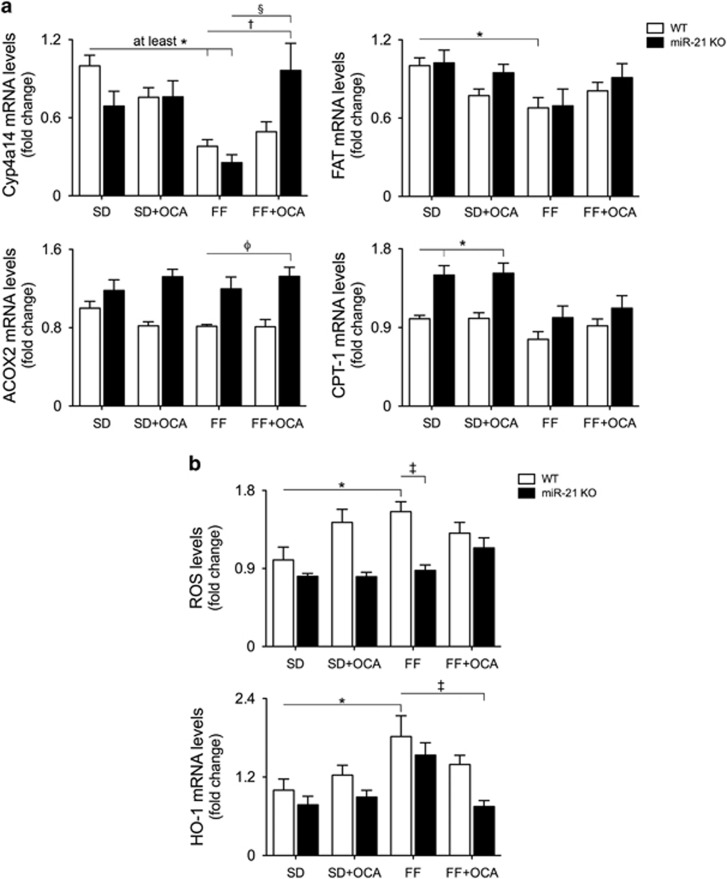
Expression of metabolism-relevant transcriptional targets of PPAR*α* is restored upon dual nuclear receptor activation. C57BL/6 WT and miR-21 KO mice were fed a FF diet (*n*=12) or a control diet (SD; *n*=12), with or without supplementation with OCA (10 mg/kg/day) for 25 weeks. (**a**) qRT-PCR analysis of Cyp4a14, FAT, ACOX-2 and CPT-1. (**b**) ROS levels (top) and qRT-PCR analysis of HO-1 (bottom). Results are expressed as mean±S.E.M. fold change. *P<0.05 and ***P<0.001 from WT control mice; ^†^*P*<0.05, ^‡^*P*<0.01 and ^*ϕ*^*P*<0.001 compared with WT FF-fed mice. SD, standard diet

In parallel with PPAR*α*, we next evaluated the expression of some of its key metabolic transcriptional targets. mRNA levels of Cyp4a14 and of genes involved in fatty acid transport and *β*-oxidation were significantly downregulated in WT mice challenged with a FF diet (*P*<0.05), whereas the dual activation of PPAR*α* and FXR resulted in complete reversion to control levels (*P*<0.05; Figure 3a). For an unbiased analysis of lipid and metabolic profiles, we further evaluated the expression of 44 mouse lipid-regulated genes using a TaqMan Array (Table 2). In WT FF-fed mice, mRNA levels of genes coding for proteins involved in cholesterol (ABCA1 and ABCG1) and fatty acid transport (FABP4, FABP5 and SLC16A6) were strongly induced, between 1.3- and 10.6-fold. Conversely, cholesterol synthesis, as revealed by the expression levels of HmgCoA synthase and STARD4, was repressed to 0.5- and 0.8-fold in WT FF-fed mice, respectively, possibly mirroring a negative feedback to the increased cholesterol uptake by the liver. Moreover, unsaturated fatty acids biosynthesis was strongly induced by the FF diet, as evidenced by increased expression levels of Alox5 (20.-fold), Alox5ap (5.2-fold), Alox12 (9.2-fold) and FADS3 (2.2-fold), along with an increase in lipase LPL (7.2-fold) and PLA2 (8.9-fold). Mitochondrial *β*-oxidation was severely compromised in WT FF-fed mice, as the expression levels of ACAT1, VLCAD and HADHB enzymes were markedly reduced. In addition, pro-inflammatory cytokines TNF-*α*, COX-2, IL-1*β* and IL-6 were strongly induced (6.5-, 5.6- and 12.6-fold, respectively). In all these settings, both miR-21 absence and FXR activation by OCA counteracted the effects of the FF diet. Of note, almost complete reversion of these changes was observed only in miR-21 KO FF+OCA-fed mice. Array results were confirmed/validated through independent real-time RT-PCR of select genes (Supplementary Figure S2).

Excessive accumulation of fatty acids within hepatocytes and high expression levels of TNF-*α* result in increased production of ROS, culminating in hepatocyte toxicity, inflammation and fibrosis.^26, 27^ Indeed, WT FF-fed mice displayed a striking increase in UCP2 expression, a mitochondrial uncoupling protein that controls mitochondrial-derived ROS, whereas miR-21 KO FF+OCA-fed mice showcased decreased levels (Table 2). Concomitantly, ROS production increased by ~50% in WT FF-fed animals (*P*<0.05) but was completely abrogated in miR-21 KO animals (*P*<0.05). OCA alone slightly increased ROS production; however, ROS levels were maintained at basal levels in miR-21 KO FF+OCA-fed mice (Figure 3b top). In response to increased oxidative stress in WT FF-fed mice, liver mRNA levels of inducible enzyme heme oxygenase-1 (HO-1) increased by almost twofold (*P*<0.05 *versus* SD-fed mice), whereas its expression in miR-21 KO FF+OCA-fed mice was similar to WT controls (*P*<0.01 *versus* FF-fed mice; Figure 3b bottom).

### Hepatic insulin sensitivity is restored upon simultaneous activation of PPAR*α* and FXR

**Figure 4:**
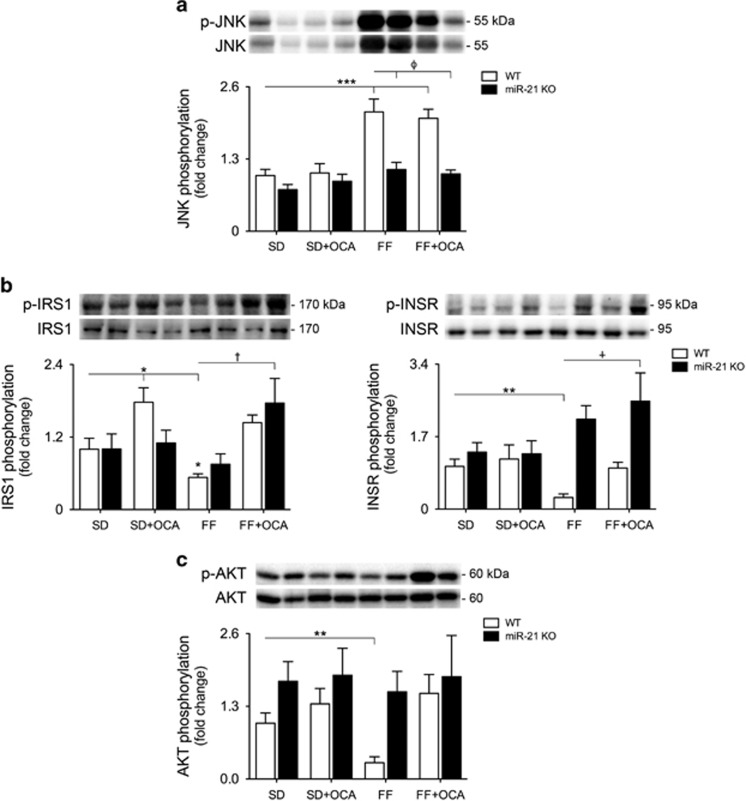
Hepatic insulin sensitivity is restored upon PPAR*α* and FXR activation. C57BL/6 WT and miR-21 KO mice were fed a FF diet (*n*=12) or a control diet (SD; *n*=12), with or without supplementation with OCA (10 mg/kg/day) for 25 weeks. (**a**) Immunoblotting of p-JNK. Representative blots are shown. Blots were normalized to total JNK. (**b**) Immunoblotting of p-IRS1 (left) and p-INSR (right). Representative blots are shown. Blots were normalized to total IRS1 and INSR respectively. (**c**) Immunblotting of p-AKT. Representative blots are shown. Blots were normalized to total AKT. Results are expressed as mean±S.E.M. fold change. *P<0.05 and **P<0.01 from WT control mice; ^†^*P*<0.05 and ^*ϕ*^*P*<0.001 compared with WT FF-fed mice. SD, standard diet

Hepatic insulin resistance is a common feature of NAFLD, even in non-obese patients.^28^ As such, we next evaluated protein levels of insulin signaling mediators in the liver of mice challenged with the FF model. JNK phosphorylation was significantly increased by ~2-fold in WT mice challenged with FF diet (*P*<0.05), whereas FXR and PPAR*α* dual activation counteracted JNK phosphorylation (Figure 4a). Curiously, total JNK was also increased by the obesogenic diet, likely as a consequence of sustained increases in lipid and cholesterol levels, decreasing in OCA-fed- or miR-21 KO- FF-fed mice. Insulin receptor (INSR) and insulin receptor substrate 1 (IRS-1) phosphorylation were reduced by 70% and 50%, respectively, in WT FF-fed mice. Supplementation of diets with OCA increased insulin signaling in both control and obesogenic diets, whereas miR-21 KO FF+OCA-fed mice displayed fully restored insulin sensitivity in the liver (Figure 4b). In accordance with INSR and IRS-1, AKT phosphorylation was significantly reduced (*P*<0.05) in WT FF-fed mice, co-substantiating insulin resistance. Of note, these effects were completely abrogated in miR-21 KO FF+OCA-fed mice (Figure 4c), further corroborating the re-establishment of insulin sensitivity.

### miR-21 and insulin resistance are increased in the skeletal muscle of mice with NASH

**Figure 5:**
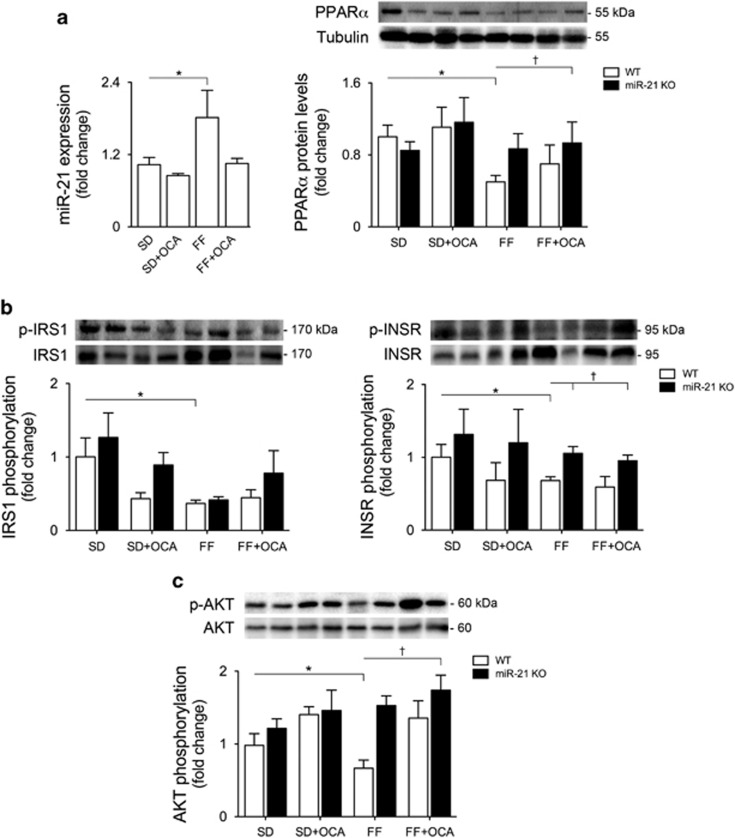
The miR-21/PPAR*α* axis is modulated in the muscle of mice with NASH, and muscle insulin resistance is prevented upon miR-21 ablation and FXR activation. C57BL/6 WT and miR-21 KO mice were fed a FF diet (*n*=12) or a control diet (SD; *n*=12), with or without supplementation with OCA (10 mg/kg/day) for 25 weeks. (**a**) qRT-PCR analysis of miR-21 (left) and immunoblotting of PPAR*α* (right). Representative blots are shown. Blots were normalized to endogenous tubulin. (**b**) Immunoblotting of p-IRS1 (left) and p-INSR (right). Representative blots are shown. Blots were normalized to total IRS1 and INSR, respectively. (**c**) Immunblotting of p-AKT. Representative blots are shown. Blots were normalized to total AKT. Results are expressed as mean±S.E.M. fold change. *P<0.05 and **P<0.01 from WT control mice; ^†^*P*<0.05 compared with WT FF-fed mice. SD, standard diet

We have previously shown that, in morbidly obese patients with NASH, insulin resistance is more pronounced in the muscle tissue comparing with the liver, and strongly correlates with disease severity. In turn, adipose tissue insulin resistance is very low.^23^ Interestingly, miR-21 expression levels in both visceral and sub cutaneous adipose tissues were not modulated by the FF diet (Supplementary Figure S3). However, miR-21 increased by almost twofold in the muscle of WT FF-fed mice (*P*<0.05), reverting to control levels in WT FF+OCA-fed mice (Figure 5a, left). PPAR*α* protein levels showed an inverse pattern (Figure 5a, right). In addition, INSR-, Akt- and IRS-1 tyrosine-phosphorylation levels decreased in the muscle of WT FF-fed mice (*P*<0.05), whereas insulin signaling was partially restored in miR-21 KO FF+OCA-fed mice (Figures 5b and c). Overall, the dual activation of PPAR*α* and FXR appears to completely prevent FF-induced insulin resistance not only in the liver, but also in skeletal muscle, further highlighting the potential of this prospective therapeutic approach.

### miR-21 is increased in liver, skeletal muscle and serum of NAFLD patients

**Figure 6:**
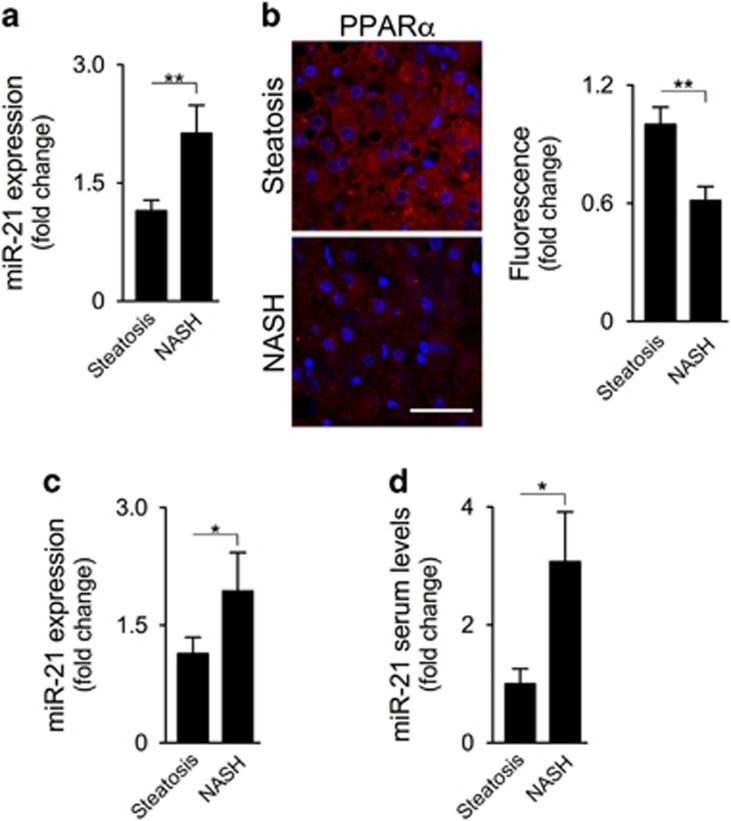
miR-21 expression is increased in the liver, muscle and serum of NAFLD patients. (**a**) qRT-PCR analysis of liver miR-21. (**b**) Representative PPAR*α* immunostaining (red) in liver tissue from patients. Nuclei were counterstained with *Hoechst* 33258 (blue). The corresponding histogram shows the quantification of PPAR*α* mean fluorescence intensity, as described in Materials and Methods section. At least five images per liver sample were used. Scale bar=10 *μ*m. (**c**) qRT-PCR analysis of muscle miR-21. (**d**) qRT-PCR analysis of serum miR-21. Results are expressed as mean±S.E.M. fold change. *P<0.05 and **P<0.01 from steatosis

Corroborating the results obtained with the animal models of NASH, expression of miR-21 in the liver of NAFLD patients significantly increased from steatosis to NASH (*P*<0.01; Figure 6a), with a concomitant decrease in PPAR*α* expression (*P*<0.01; Figure 6b). miR-21 was similarly modulated in the muscle, significantly increasing in patients with NASH when compared with those with simple steatosis (*P*<0.05; Figure 6c). Finally, we also evaluated the potential of miR-21 as a serum biomarker. We found that levels of miR-21 significantly increased from steatosis to NASH by ~3-fold (*P*<0.05; Figure 6d), substantiating the relevance of this miRNA in NAFLD pathogenesis.

## Discussion

Along with others, we have shown that miRNAs are deregulated in animal models of steatohepatitis and human NASH, thus contributing for disease pathogenesis.^6, 21, 29, 30, 31^ Using complementary animal models of NASH, our present study shows that miR-21 ablation significantly reduces liver steatosis, inflammation and fibrosis. For the first time, we demonstrated that hepatocyte lipoapoptosis, insulin resistance, and overall lipid and cholesterol metabolism are strongly improved in miR-21 KO animals. These effects may largely be attributed to PPAR*α*^6^ although additional direct and indirect miR-21 targets are conceivable. Importantly, we also showed that by combining miR-21 ablation with activation of FXR, through the use of OCA, NASH development is completely inhibited, with normalization of several features of the metabolic syndrome. We further demonstrate that miR-21 is increased not only in the liver but also in skeletal muscle of mouse and human NASH, further corroborating its involvement in disease pathogenesis.

Our results showed that steatosis and inflammation progressively increase with time after MCD feeding, in parallel with a progressive decrease in PPAR*α*. Surprisingly, miR-21 did not increase from 2 to 8 weeks of MCD feeding, inversely to the gradual decrease in PPAR*α*. Thus, PPAR*α* may not be solely regulated by miR-21 during disease progression. For instance, miR-10b and miR-34a have been shown to regulate cellular steatosis by targeting PPAR*α*.^32, 33^ Still, miR-21 ablation ameliorated hepatic steatosis, liver cell injury, inflammation and fibrogenesis, particularly at 8 weeks of feeding, suggesting that miR-21 has a major role in NAFLD progression, rather than in disease triggering.

Active caspase-2 has been found increased in both mouse and human NASH and is essential for induction of lipoapoptosis,^34^ whereas its inhibition prevents steatosis and fibrosis.^22^ Our results show, for the first time, that miR-21 ablation prevents caspase-2 activation and significantly impairs lipoapoptosis. We have recently shown that deoxycholic acid-induced oxidative stress in hepatocytes results in caspase-2 activation and NF-*κ*B/miR-21 inhibition, in a PIDD-dependent manner.^35^ Still, the major effect of miR-21 ablation in preventing caspase-2 activation, apoptosis and fibrosis may result from the noticeable decrease in lipid accumulation. In addition, histological analysis also revealed a marked decrease in liver necrotic areas in the absence of miR-21. Considering that miR-21 was recently reported to induce regulated necrosis in mice^36^ and because we have shown that necroptosis associates with the development of NASH and fibrosis,^18^ miR-21 may further contribute to fibrogenesis by activating necroptosis.

The simultaneous activation of distinct metabolic pathways may prove to be a valuable strategy to treat NASH. For instance, PLA2 ablation or pharmacological inhibition alleviates fat-induced liver damage and fibrosis^37, 38^ and we show that FF-induced PLA2 and LPL lipases revert to control levels upon activation of both nuclear receptors. NASH-associated fibrosis also results from activation of Kupfer and stellate cells, downstream of cholesterol accumulation,^39^ and miR-21 KO FF+OCA-fed mice displayed a complete reversion of hepatic cholesterol content, along with inhibition of Alox gene family expression. In fact, lipid polyunsaturation and the Alox5 pathway associate with obesity, insulin resistance and NAFLD.^40^ Altogether, we showed that simultaneous activation of PPAR*α* and FXR robustly modulates hepatic lipid metabolism, resulting in a strong reduction in steatosis and cholesterol accumulation by decreasing fatty acid uptake in the liver, impairing metabolic pathways involved in fatty acid polyunsaturation, and re-establishing *β*-oxidation to basal levels. Importantly, this also correlated with body weight reduction and prevention of obesity and hepatomegaly, much more evident in OCA-fed mice.

In obesity, fatty acids accumulate in the liver and skeletal muscle, saturating their oxidative capacity,^41, 42^ and exacerbating insulin resistance. In agreement, we show that upon an obesogenic diet, the miR-21/PPAR*α* axis is impaired not only in the liver but also in skeletal muscle, likely resulting from intramyocellular lipid accumulation. Of note, insulin resistance was completely prevented only in miR-21 KO FF+OCA-fed mice. In addition, FF-induced oxidative stress, mitochondrial dysfunction and HO-1 expression were completely abrogated in miR-21 KO FF+OCA-fed mice, possibly as a response to the re-established lipid metabolism. Alternatively, PPAR*α* may have a role in regulating insulin sensitivity; it was recently shown that miR-34a inhibition ameliorates NASH by targeting the PPAR*α*/AMPK pathway, thus regulating insulin signaling and steatosis.^33^ Altogether, restoring mitochondrial function appears to be key in counteracting metabolic overload and reinstating insulin signaling. Supporting this notion, miR-21 inhibition ameliorates Alport nephropathy by stimulating metabolism and supporting mitochondrial function,^43^ whereas FXR was shown to also regulate mitochondrial function and exert anti-oxidant effects.^44^

Finally, we showed that miR-21 is increased in both liver and muscle of morbidly obese NASH patients, concomitantly with decreased PPAR*α* levels. Of note, previous studies from our group have shown that serum levels of cholesterol and triglycerides are increased in patients with NASH, compared with simple steatosis.^23^ Therefore, a possible inverse correlation between these serum determinations and hepatic PPAR*α* levels may be established. In addition, serum miR-21 levels markedly increased with disease severity, highlighting its potential as a noninvasive biomarker of NASH.^29^

Several therapeutic approaches for NASH are currently under evaluation including PPAR*α* and FXR agonists.^45^ Results from the FLINT trial have highlighted OCA as a promising therapeutic agent in NASH^17^ and Mudaliar *et al.*^46^ have documented its efficacy and safety in the treatment of patients with NAFLD and diabetes. Still, treatment with OCA has been associated with greater hepatic insulin resistance; an increase in average total cholesterol and LDL; and a decrease in average HDL.^17^ Elafibranor, a dual PPAR*α/δ* agonist, is also being studied in animal models of NASH, and was shown to improve steatosis, inflammation and fibrosis.^47^ Nevertheless, this agonist mainly acts on the liver, with little or no effects in the muscle,^47^ and elicits several side effects.^45^ The ongoing phase 3 study will be important to ascertain the precise effects of elafibranor.^45^ Finally, insulin resistance is much more prominent in the muscle of NASH patients, rather than in the liver or adipose tissues,^23^ and strongly contributes to disease pathogenesis. Therefore, a therapy targeting the different metabolic organs affected in NAFLD, particularly the liver and the muscle, may constitute a milestone in finding a treatment for NASH. Given the state-of-the-art on anti-miR-122 for the treatment of hepatitis C,^48^ and despite the need to identify any potential undesired effects of antagonizing miR-21 expression in a chronic manner, the use of antagomiR-21 combined with OCA for the treatment of NASH is an interesting therapeutic strategy that deserves further investigation.

## Materials and methods

### Animals, diets and sample collection

WT and miR-21 KO 5-month old C57BL/6N male mice were fed either a control diet (*n*=5) or a MCD (*n*=18) diet (TestDiet, St. Louis, MO, USA) for 2 and 8 weeks. In parallel, 10-weeks old C57BL/6N WT and miR-21 KO mice were fed either a SD (*n*=12) or a FF diet^25^ (*n*=12) for 25 weeks. Six animals from each group had their diet supplemented with OCA 10 mg/kg/day (provided by Intercept Pharmaceuticals, Inc., New York, NY, USA) and both males and females were included in all groups. Animal protocols, histopathology and serum analysis were performed as described in Supplementary Materials and Methods. All animals received humane care according to the criteria outlined in the 'Guide for the Care and Use of Laboratory Animals' prepared by the National Academy of Sciences and published by the National Institutes of Health (NIH publication 86-23 revised 1985).

### Quantitative RT-PCR (qPCR), total protein extraction and immunoblotting

RNA extraction and qPCR analysis, as well as total protein extraction and immunoblotting were performed as described in Supplementary Materials and Methods.

### TUNEL assay

TUNEL assay was performed in 4 *μ*M liver sections to detect and quantify apoptosis, using the ApopTag Red *In Situ* Apoptosis Detection Kit (Merck Millipore, Darmstadt, Germany), according to manufacturer's instructions (Supplementary Materials and Methods).

### Immunohistochemistry and image analysis

PPAR*α* expression was evaluated by immunohistochemistry in paraffin-embedded liver sections from morbid obese patients with NAFLD as detailed in Supplementary Materials and Methods.

### Total ROS levels measurement

2'-7'-Dichlorodihydrofluorescein diacetate (H_2_DCFDA; Sigma-Aldrich Co., St. Louis, MO, USA) was used as a cell-permeant non-fluorescent molecule that is oxidized by ROS to form dichlorofluorescein, a fluorescent compound, as described in Supplementary Materials and Methods.

### Cholesterol measurement

Liver cholesterol contents were measured using the Cholesterol/Cholesteryl Ester Quantification Assay Kit (ab65359, Abcam plc, Cambridge, UK), following manufacturer's instructions. Fluorescence emission was measured using the GloMax-Multi+ Detection system (Promega Corp., Madison, WI, USA).

### Lipid-regulated genes array

A Taqman qPCR Array, containing 44 genes involved in sterol and fatty acid metabolism was performed to evaluate the expression of mouse lipid regulated genes (#4415461, Thermo Fisher Scientific, Waltham, MA, USA), as described in Supplementary Materials and Methods.

### Patients and histology

NASH liver (*n*=28) and muscle (*n*=28) specimens were obtained from morbidly obese patients undergoing bariatric surgery, as previously described.^23^ No statistical differences were observed in age and gender between patient groups. Liver and muscle biopsies were processed for total protein and RNA isolation. Paraffin-embedded liver tissue sections from NAFLD patients were stained as described in Supplementary Materials and Methods and blindly evaluated by an experienced pathologist. Informed written consent was obtained from all patients and the study protocol conformed to the Ethical Guidelines of the 1975 Declaration of Helsinki, as reflected in *a priori* approval by the Hospital of Santa Maria (Lisbon, Portugal) Human Ethics Committee.

### Serum miRNA extraction and quantification

Total RNA was isolated from 100 *μ*l of serum from NAFLD patients (*n*=24) using the miRCURY RNA Isolation Kit (Exiqon, Woburn, MA, USA), following the manufacturer's instructions, and processed for miRNA quantification as described in Supplementary Materials and Methods.

### Densitometry and statistical analysis

The relative intensities of protein bands were analyzed using ImageLab Version 5.1 densitometric analysis program (Bio-Rad Laboratories, Hercules, CA, USA). Statistical analysis was performed using GraphPad Prism version 5.0 (GraphPad Software, San Diego, CA, USA). One-way analysis of variance (ANOVA) test was used for comparisons when more than two groups were analyzed and Student's *t*-test when two groups were analyzed. ANOVA was performed using the Bonferroni *post-hoc* test. Values of *P*<0.05 were considered significant.

## Figures and Tables

**Table 1:** Histological data of the MCD mouse model

	**2 Weeks**			**8 Weeks**		
	**Control**	**MCD**		**Control**	**MCD**	
	**WT (*n*=5)**	**WT (*n*=10)**	**miR-21 KO (*n*=8)**	**WT (*n*=5)**	**WT (*n*=10)**	**miR-21 KO (*n*=8)**
*Steatosis*						
0 (None)	80%		12,5%	100%		
1 (Minimal)	20%	10%***	12,5%			
2 (Mild)		80%	50%		22%***	42%
3 (Moderate)		10%	25%		45%	29%
4 (Severe)					33%	29%

*Lobular inflammation*						
0 (None)	80%	40%	25%	80%	33%*	14%
1 (Mild)	20%	50%	25%	20%	11%	57%
2 (Moderate)			50%			28%
3 (Severe)		10%			56%	

*Necrosis*						
0 (None)	100%	80%	33%	100%	33%*	29%
1 (Mild)		20%	67%		11%	42%
2 (Moderate)						29%
3 (Severe)					56%	

*Pericellular fibrosis*						
0 (None)	100%	100%	100%	100%	10%***	86%^‡^
1 (Mild)					80%	14%
2 (Moderate)					10%	
3 (Severe)						

*Hypertrophy*						
0 (None)	100%	50%	62%	100%	44%*	43%
1 (Mild)		40%				43%
2 (Moderate)		10%	38%			14%
3 (Severe)					56%	

Histological features are presented as percentage of cases in each grade

*P<0.05 and ***P<0.001 from respective WT control-fed mice. ^‡^*P*<0.01 from WT MCD-fed mice

**Table 2:** Expression of lipid regulated genes in the livers of mice from the FF model

	**Gene**	**WT**			**miR-21 KO**		
		**SD**	**FF**	**FF+OCA**	**SD**	**FF**	**FF+OCA**
Cholesterol transport and synthesis	**ABCA1**	**1.0**	**1.3**	**1.3**	**1.3**	**1.2**	**1.1**
	**ABCG1**	**1.0**	**6.4**	**3.3**	**1.6**	**2.3**	**2.1**
	SOAT2	1.0	1.4	1.8	2.3	1.9	2.2
	Apoe	1.0	0.9	1.2	1.6	1.3	1.2
	Cyp27a1	1.0	1.1	0.9	1.0	0.9	0.8
	HMGCR	1.0	1.3	3.7	1.4	1.4	1.3
	**HMGCS1**	**1.0**	**0.5**	**1.5**	**1.4**	**0.5**	**0.6**
	Insig1	1.0	1.1	2.7	2.2	1.5	1.8
	LDLR	1.0	0.9	2.3	1.8	1.4	1.8
	SREBF2	1.0	1.0	1.8	1.3	0.8	1.1
	**STARD4**	**1.0**	**0.8**	**2.2**	**1.5**	**1.1**	**1.7**
	Tbxas	1.0	5.9	4.2	2.3	2.8	2.2
Fatty acid metabolism and *β*-oxidation	**ACAT1**	**1**.**0**	**0.6**	**0.9**	**1.4**	**1.2**	**1.4**
	**Alox12**	**1.0**	**9.2**	**3.8**	**2.4**	**3.1**	**3.5**
	Alox15	1.0	0.9	0.6	8.3	2.6	0.7
	**Alox5**	**1.0**	**20.0**	**9.0**	**4.4**	**4.1**	**2.2**
	**Alox5ap**	**1.0**	**5.2**	**2.0**	**1.9**	**2.4**	**1.8**
	CD36	1.0	4.1	5.8	6.3	4.9	3.2
	FADS1	1.0	0.7	1.3	1.6	1.1	1.2
	FADS2	1.0	0.7	2.0	1.6	1.6	2.0
	FADS3	1.0	2.2	2.0	1.5	1.0	0.9
	Gyk	1.0	0.9	0.8	0.9	1.0	0.8
	**HADHB**	**1.0**	**0.7**	**11.2**	**3.3**	**13.8**	**1.1**
	LTA4H	1.0	0.9	1.0	1.3	1.0	0.9
	LTC4S	1.0	0.7	0.5	1.3	0.9	1.0
	**LPL**	**1.0**	**7.2**	**3.3**	**1.4**	**1.8**	**1.4**
	LXR	1.0	1.3	1.3	1.5	1.2	1.1
	**PLA2**	**1.0**	**8.9**	**5.5**	**1.8**	**3.3**	**1.8**
	PPAR*δ*	1.0	2.1	2.1	1.7	2.0	2.1
	PPAR*γ*	1.0	2.0	2.7	2.7	2.3	1.1
	SREBF1	1.0	1.4	2.5	2.6	5.1	3.7
	SCD1	1.0	0.9	0.5	1.0	1.0	0.2
	**UCP2**	**1.0**	**9.0**	**5.6**	**3.4**	**3.1**	**3.5**
	**VLCAD**	**1.0**	**0.8**	**0.9**	**1.1**	**1.0**	**1.1**
Fatty acid transport	**FABP4**	**1.0**	**7.9**	**4.20**	**2.5**	**2.1**	**1.3**
	**FABP5**	**1.0**	**10.6**	**3.69**	**2.9**	**3.0**	**3.7**
	**SLC16A6**	**1.0**	**2.5**	**1.93**	**3.6**	**1.3**	**1.2**
	SLC27A1	1.0	0.5	0.64	0.9	0.5	0.7
	SLC27A3	1.0	1.2	1.93	2.0	1.5	1.2
Inflammation	**IL-1*****β***	**1.0**	**5.6**	**3.93**	**2.45**	**2.27**	**1.8**
	**IL-6**	**1.0**	**12.6**	**6.43**	**4.41**	**4.07**	**2.4**
	**COX-2**	**1.0**	**3.5**	**3.07**	**2.41**	**0.95**	**0.9**
	**TNF-*****α***	**1.0**	**6.5**	**4.00**	**2.15**	**1.57**	**0.9**

Total RNA from four animals on each of the groups from the FF model were combined and used in the Taqman array to evaluate the expression of lipid-regulated genes. Results are expressed in fold change and the most relevant genes are presented in bold. SD, standard diet
