# miR-21 ablation and obeticholic acid ameliorate nonalcoholic steatohepatitis in mice

**DOI:** 10.1038/cddis.2017.246

**Published:** 2017-05-25

**Authors:** Pedro M Rodrigues, Marta B Afonso, André L Simão, Catarina C Carvalho, Alexandre Trindade, António Duarte, Pedro M Borralho, Mariana V Machado, Helena Cortez-Pinto, Cecília MP Rodrigues, Rui E Castro

**Correction to:** *Cell Death and Disease* (2017) **8**, e2748; doi:10.1038/cddis.2017.172; published online 13 April 2017

Since the publication of this paper, it has been noted that Figure 1 was split to appear in two different pages resulting in panels (a)–(g) being displaced, rendering the figure incomprehensible.

The figure has been reformatted to appear on one page.

The corrected article appears online together with this erratum. The publishers apologize for any inconvenience this may have caused.

