# Supplementary material for: miR-21 ablation and obeticholic acid ameliorate nonalcoholic steatohepatitis in mice
Source: Cell Death Dis. 2017 Apr 13;8(4):e2748–. doi: 10.1038/cddis.2017.172 (PMC5477590; doi:10.1038/cddis.2017.172)
Supplement: Supplementary Information [file cddis2017172x1.pdf]

Supplementary materials and methods

Animals and sample collection

miR-21 KO mice were generated as previously described (Patrick *et al.,* The Journal of Clinical Investigation 2010; DOI: 10.1172/JCI43604). Briefly loxP sites were introduced at both ends of pre-miR-21 through homologous recombination. Global deletion of miR-21 was achieved by breeding Mir21fl/+ mice to mice expressing CAG-Cre. miR-21 KO mice are viable and fertile, displaying no overt abnormalities. Similarly, mutant mice display no abnormalities in liver size.

Animals were weighed once a week before sacrifice by exsanguination under isoflurane anaesthesia. Cardiac perfusion was performed with cold Phosphate Buffered Saline (PBS) 1X and one liver lobe, muscle and visceral adipose tissue were removed, rinsed in normal saline, flash frozen in liquid nitrogen and stored at -80 ºC for protein and RNA extraction. Another liver lobe was included in optimal cutting temperature compound (Tissue-Tek OCT; 4583, Sakura Finetek Europe B.V., The Netherlands) for histochemistry of fat by Oil Red O staining (O-0625, Sigma-Aldrich Co., St Louis, MO, USA). Paraffin-embedded sections (3-4 µm) were stained with hematoxylin and eosin (H&E) or Masson's Trichrome. Liver sections were blinded scored by an experienced pathologist, using a four-point severity scale (0, normal; 1, mild; 2, moderate; 3, severe) for steatosis, inflammation and fibrosis. Serum was also collected for alanine aminotransferase (ALT) determination, using standard clinical chemistry techniques. The experimental protocol was approved by Direção Geral de Alimentação e Veterinária. Animals received humane care in a temperature-controlled environment with a 12-h light–dark cycle, complying with the Institute's guidelines, and with the permission of the local animal ethical committee in accordance with the EU Directive (2010/63/EU), Portuguese

law (DL 113/2013), and all relevant legislation. All experiments were performed by an investigator accredited for directing animal experiments (FELASA level C).

Quantitative RT-PCR (qPCR)

Total RNA was extracted using the TRIzol® reagent according to the manufacturer's instructions (Thermo Fisher Scientific, Waltham, MA USA). Real-Time RT-PCR was performed in an Applied Biosystems 7300 system (Thermo Fisher Scientific) to quantitate the expression of miR-21. U6 snRNA was used as the normalization control. The relative amount of miR-21 was determined by the threshold cycle ($2^{-\Delta\Delta CT}$) method, where $\Delta\Delta C_T = (C_{TmiR\text{-}21} - C_{TU6})$ sample – $(C_{TmiR\text{-}21} - C_{TU6})$ calibrator. To assess the expression levels of other genes, the primer sequences displayed in Table S1 were used. Two independent reactions for each primer set were assessed in a total volume of 12.5 µl containing 2x Power SYBR green PCR master mix (Thermo Fisher Scientific) and 0.6 µM (each) primer. The relative amounts of each gene were calculated based on the standard curve and were normalized to the level of hypoxanthine phosphoribosyltransferase (HPRT) and expressed as fold change from controls.

Total protein extraction

Liver and muscle pieces were homogenized in ice-cold lysis buffer (10 mM Tris/HCl, pH 7.6, 5 mM $MgCl_2$, 1.5 mM potassium acetate, 1% Nonidet p-40. 2 mM DTT) and 1X Halt Protease and Phosphatase Inhibitor Cocktail (Thermo Fisher Scientific) using a dounce homogenizer. Afterwards, the lysate was centrifuged at 10 000 *g* for 10 min at 4ºC and the supernatant was recovered and stored at -80ºC. To determine protein concentration, the Bio-Rad protein assay kit (Bio-Rad Laboratories, Hercules, CA, USA) was used, according to the manufacturer's specifications.

Immunoblotting

Fifty micrograms of total protein extracts were separated on a 6 or 10% sodium dodecyl sulphate-polyacrilamide gel electrophoresis (SDS-PAGE) and then transferred onto a nitrocellulose membrane and blocked with 5% milk solution. Blots were incubated overnight with primary rabbit polyclonal antibodies against PPARα (sc-9000); insulin receptor (INSR; sc-711); phosphorylated INSR $Tyr^{1162/1163}$ (sc-25103); insulin receptor substrate 1 (IRS1; sc-7200); phosphorylated IRS1 Tyr^{632} (sc-17196); phosphorylated AKT Ser^{473} (sc-7985-R); and AKT (sc-8312; Santa Cruz Biotechnology, Santa Cruz, CA, USA) or primary mouse antibody against β-actin or tubulin and finally, with secondary antibodies conjugated with horseradish peroxidase (Bio-Rad Laboratories) for 3 h at room temperature. Membranes were processed for protein detection using Pierce Super Signal substrate (Thermo Fisher Scientific).

Terminal deoxynucleotidyl transferase dUTP nick end labeling (TUNEL) assay

TUNEL assay was performed in 4 μM liver sections to detect and quantify apoptosis, using the ApopTag Red *In Situ* Apoptosis Detection Kit (Merck Millipore, Darmstadt, Germany), according to manufacturer's instructions. Liver sections were counterstained with 5 μg/mL Hoechst 33258 (Sigma-Aldrich Co.) for 10 minutes at room temperature. Samples were mounted using Fluoromont-G (Beckamn Coulter, Brea, CA, USA). Detection of TUNEL-positive nuclei was performed using a AxioScope A1 fluorescent microscope (Carl Zeiss Microscopy GmbH, Jena, Germany) with a magnification of 400x. Apoptotic frequency was measured in liver sections displaying similar cell density and results are expressed as the mean number of TUNEL-positive cells per field.

Immunohistochemistry and image analysis

Liver sections were deparaffined, rehydrated and boiled three times in 10 mM citrate (pH 6.0) and then incubated for 1 h in blocking buffer, containing 0.3% Triton X-100 (Sigma-Aldrich Co.), 1% FBS (Thermo Fisher Scientific), and 10 % normal donkey serum (Jackson ImmunoResearch Laboratories, West Grove, PA, USA). Primary antibody reactive to PPARα (ab8934; Abcam plc, Cambridge, UK) was incubated overnight at 4ºC and developed by incubating a secondary DyLight 594-conjugated anti-rabbit antibody (1:200; Jackson ImmunoResearch Laboratories) for 2 h at room temperature. Cell nuclei were stained with Hoechst 33258 (Sigma-Aldrich Co.) at 50 μg/ml in PBS for 10 min at room temperature, protected from light. Samples were mounted using Fluoromount-G (Beckman Coulter) and detection of PPARα was visualized using an AxioScope.A1 microscope (Carl Zeiss Microscopy GmbH). Images were acquired using an AxioCam HRm camera with the AxioVision software (release 4.8; Carl Zeiss Microscopy GmbH) under 400x magnification. Semi-quantitative analysis of mean fluorescence intensities of PPARα was performed using the NIH ImageJ software. Eight images per sample were obtained and converted into an 8-bit format and the background subtracted. An intensity threshold was set and kept constant for all images analysed. PPARα fluorescence intensity was normalized with the area of liver tissue per microscopic field.

Total ROS levels measurement

25 mg of liver tissue were homogenized using a glass dounce in 500 µL of ice-cold PBS. The lysate was then centrifuged at 10 000 *g* for 10 min to remove insoluble particles, and the supernatant was recovered. Fifty μl of the lysate were incubated with 10 µM H_2DCFDA at room temperature for 30 min, protected from light. The emission of green fluorescence was measured using the GloMax-Multi+ Detection System (Promega Corp., Madison, WI, USA).

Lipid Regulated Genes Array

A Taqman® qPCR Array, containing 44 genes involved in sterol and fatty acid metabolism was performed to evaluate the expression of mouse lipid regulated genes (#4415461, Thermo Fisher Scientific). Briefly, RNA from animals on each of the groups from the FF model were combined and 1.5 µg of total RNA was used to synthesize single stranded cDNA, using the High Capacity RNA-to-cDNA Kit (#4387406, Thermo Fisher Scientific), following the manufacturer's protocol. Each cDNA labeled sample was used on the array, according to the manufacturer's instructions. The Taqman® Array plate was run in an Applied Biosystems 7300 system (Life Technologies Corp.).

Patients and histology

Paraffin-embedded liver tissue sections from NAFLD patients were stained with H&E. The Gordon and Sweet's Silver Staining method was used for identification of reticular fibres; Chromotrope-Aniline Blue (CAB) for connective tissue; and Perl's Prussian Blue for iron. Steatosis was graded from 0 to 3 based on the percentage of steatotic hepatocytes (0, none; 1, <33%; 2, 33%-66%; 3, >66%). Additionally, portal and lobular inflammation and portal and lobular fibrosis were semi-quantitatively graded on a scale of 0-4 (0, absence; 1, mild; 2, moderate; 3, severe degree and 4, cirrhosis), as previously reported[24].

Serum miRNA extraction and quantification

For normalization purposes, cel-miR-39 was added to the serum prior to RNA isolation and used as an internal spike-in control. cDNA was synthesized using the Taqman Advanced miRNA assay (ThermoFisher), according to the manufacturer's instructions. miR-21 expression levels were quantified by qPCR and normalized by comparison with the spike-in RNA control, using the threshold cycle method, as described above.
