# Supplementary material for: miR-21 ablation and obeticholic acid ameliorate nonalcoholic steatohepatitis in mice
Source: Cell Death Dis. 2017 Apr 13;8(4):e2748–. doi: 10.1038/cddis.2017.172 (PMC5477590; doi:10.1038/cddis.2017.172)
Supplement: Supplementary Table [file cddis2017172x2.pdf]

Supplementary Table S1 Primer sequences used for qRT-PCR analysis in mouse liver

TNF-α	Forward: 5’-AGG CAC TCC CCC AAA AGA TG-3’<br>Reverse: 5’-TGA GGG TCT GGG CCA TAG AA-3’
IL-6	Forward: 5’-GAG GAT ACC ACT CCC AAC AGA CC-3’<br>Reverse: 5’-AAG TGC ATC ATC GTT GTT CAT ACA-3’
IL-1β	Forward: 5’-TGC CAC CTT TTG ACA GTG ATG-3’<br>Reverse: 5’-TGA TGT GCT GCT GCG AGA TT-3’
TLR4	Forward: 5’-TCC CTG CAT AGA GGT AGT TCC TA-3’<br>Reverse: 5’-CTT CAA GGG GTT GAA GCT CAG-3’
Collagen1α1	Forward: 5’-CTG ACT GGA AGA GCG GAG AG-3’<br>Reverse: 5’-GAC GGC TGA GTA GGG AAC AC-3’
TGFβ	Forward: 5′-CTG CTG ACC CCC ACT GAT AC-3’<br>Reverse: 5’-GTG AGC GCT GAA TCG AAA GC-3’
CPT1A	Forward: 5’-TGG ACC CAA ATT GCA GTG GT-3’<br>Reverse: 5’-GCA TCT CCA TGG CGT AGT AGT-3’
CYP4a14	Forward: 5’-ACC CAA AGG TGT TTG ACC CC-3’<br>Reverse: 5’-CTT GCA ATG GGG ACT GGG AT-3’
ACOX2	Forward: 5’-CTG GGA CAT GGG ACA TAC CTG-3’<br>Reverse: 5’-TAG GAC CAC AGC ATG GGT GA-3’
FAT	Forward: 5’-CGG GAG AGC AAA CTA CCT ACA-3’<br>Reverse: 5’-AAC CTC ACC TTT GGG GAC T-3’
VLCAD	Forward: 5’-CAG CGA CTT TAT GCC AGG GA-3’<br>Reverse: 5’-TGG CAG GGT CAT TCA CTT CC-3’
FABP4	Forward: 5’-TGA TGC CTT TGT GGG AAC CT-3’<br>Reverse: 5’-GCC CCG CCA TCT AGG GTT AT-3’
LXR	Forward: 5’-TGT GCG CTC AGC TCT TGT C-3’<br>Reverse: 5’-CTC CGT TGC AGA ATC AGG AGA A-3’
PLA2	Forward: 5’-AAG ACC TGG GAA GTG TGA GAA-3’<br>Reverse: 5’-GAG TGT CCA GCA TAT CGC CAA-3’
LPL	Forward: 5’-TAC AAA GTG TTC CAT TAC CAA GTC A-3’<br>Reverse: 5’-ATG AGC AGT TCT CCG ATG TCC-3’
Alox5	Forward: 5’-CCT CAA GCA GCA CAG ACG TA-3’<br>Reverse: 5’-TGA ACA GGT TCT CCA TCG CTT-3’
HMGCS	Forward: 5’-GTC TCC TTG CTT TGC TCG TTC -3’<br>Reverse: 5’-GGA CAG AGA ACT GTG GTC TCC -3’
ABCA1	Forward: 5’-CCC AGA GCA AAA AGC GAC TC-3’<br>Reverse: 5’-GGT CAT CAT CAC TTT GGT CCT TG-3’
ABCG1	Forward: 5’-AGG CAG ACG AGA GAT GGT CA-3’<br>Reverse: 5’-GAC ACC ACT TGG AAG CAG GA-3’
Srebp1c	Forward: 5’-GCA GGA GAC TGA GAG ACC CC-3’<br>Reverse: 5’-GTA CCC ACT GGC CTT CTC AC-3’
Cyp7a1	Forward: 5’-CTG GGG GAT TGC TGT GGT AG-3’<br>Reverse: 5’-GCA CAG CCC AGG TAT GGA AT-3’
SHP	Forward: 5’-CTC TTC AAC CCA GAT GTG CCA-3’<br>Reverse: 5’-CCA GGG CTC CAA GAC TTC AC-3’
HO-1	Forward: 5’-CGG GCC AGC AAC AAA GTG-3’<br>Reverse: 5’-AGT GTA AGG ACC CAT CGG AGA A-3’
HPRT	Forward: 5’-GGT GAA AAG GAC CTC TCG AAG TG-3’<br>Reverse: 5’-ATA GTC AAG GGC ATA TCC AAC AAC A-3’
