# Supplementary material for: miR-21 ablation and obeticholic acid ameliorate nonalcoholic steatohepatitis in mice
Source: Cell Death Dis. 2017 Apr 13;8(4):e2748–. doi: 10.1038/cddis.2017.172 (PMC5477590; doi:10.1038/cddis.2017.172)
Supplement: Supplementary Figure [file cddis2017172x3.docx]

**Supplementary Figures**

**Figure S1**


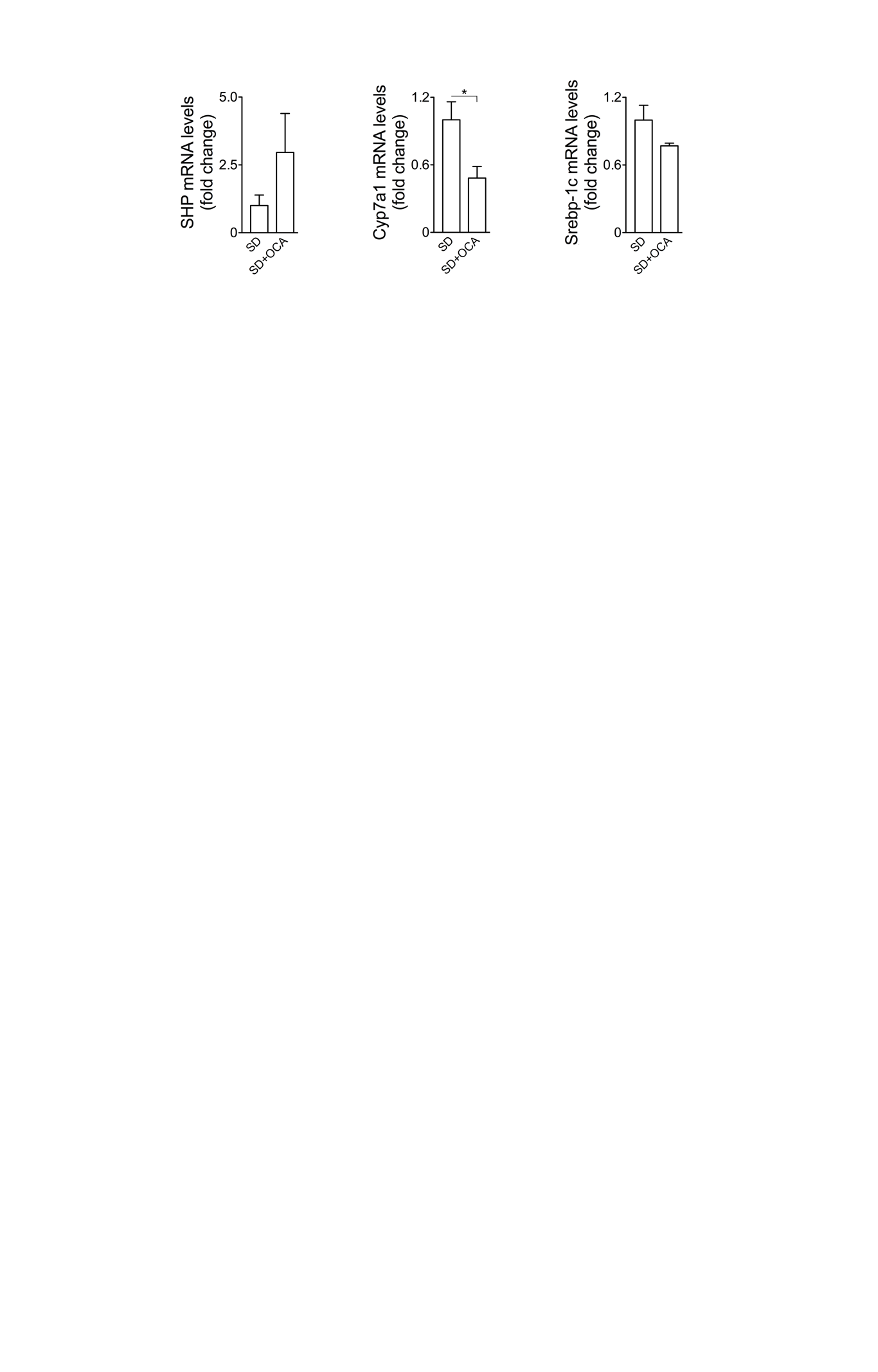


**FXR is activated by OCA.** qRT-PCR analysis of SHP (left), Cyp7a1 (middle) and Srebp-1c (right) in mouse liver. Results are expressed as mean ± SEM fold change. **p* < 0.05, compared with respective WT SD-fed mice.

**Figure S2**


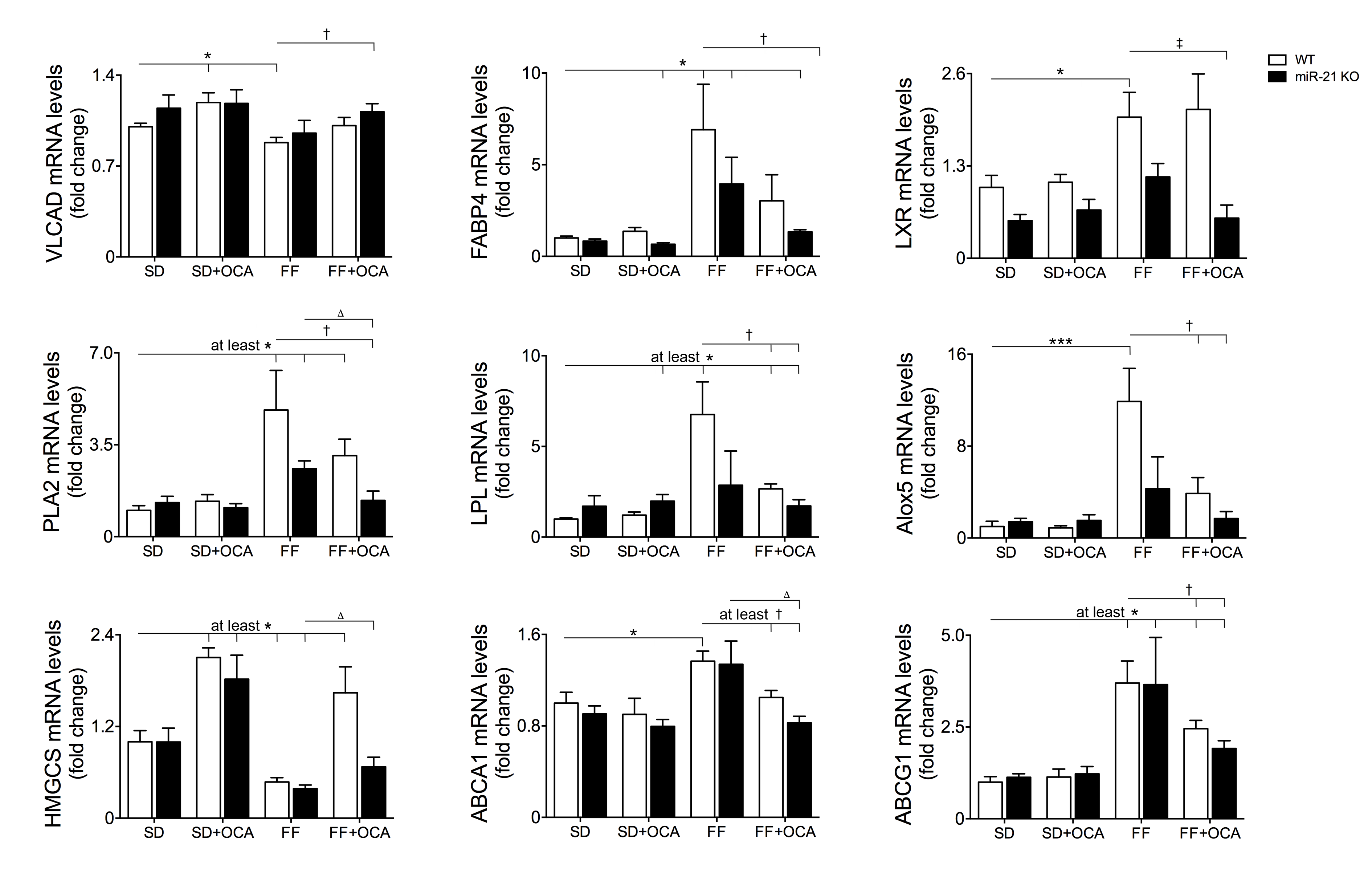


**Lipid and cholesterol metabolism mediators are modulated upon FXR and PPARα activation.** qRT-PCR analysis of VLCAD, FABP4, LXR, PLA2, LPL, Alox5, HMGCS, ABCA1 and ABCG1. Results are expressed as mean ± SEM fold change. **p* < 0.05, ***p* < 0.01 and ****p* < 0.001, compared with respective WT SD-fed mice; ^†^*p* < 0.05 and ^‡^*p* < 0.01 compared with WT FF-fed mice; ^Δ^*p* < 0.05 vs miR-21 KO FF-fed mice.

**Figure S3**


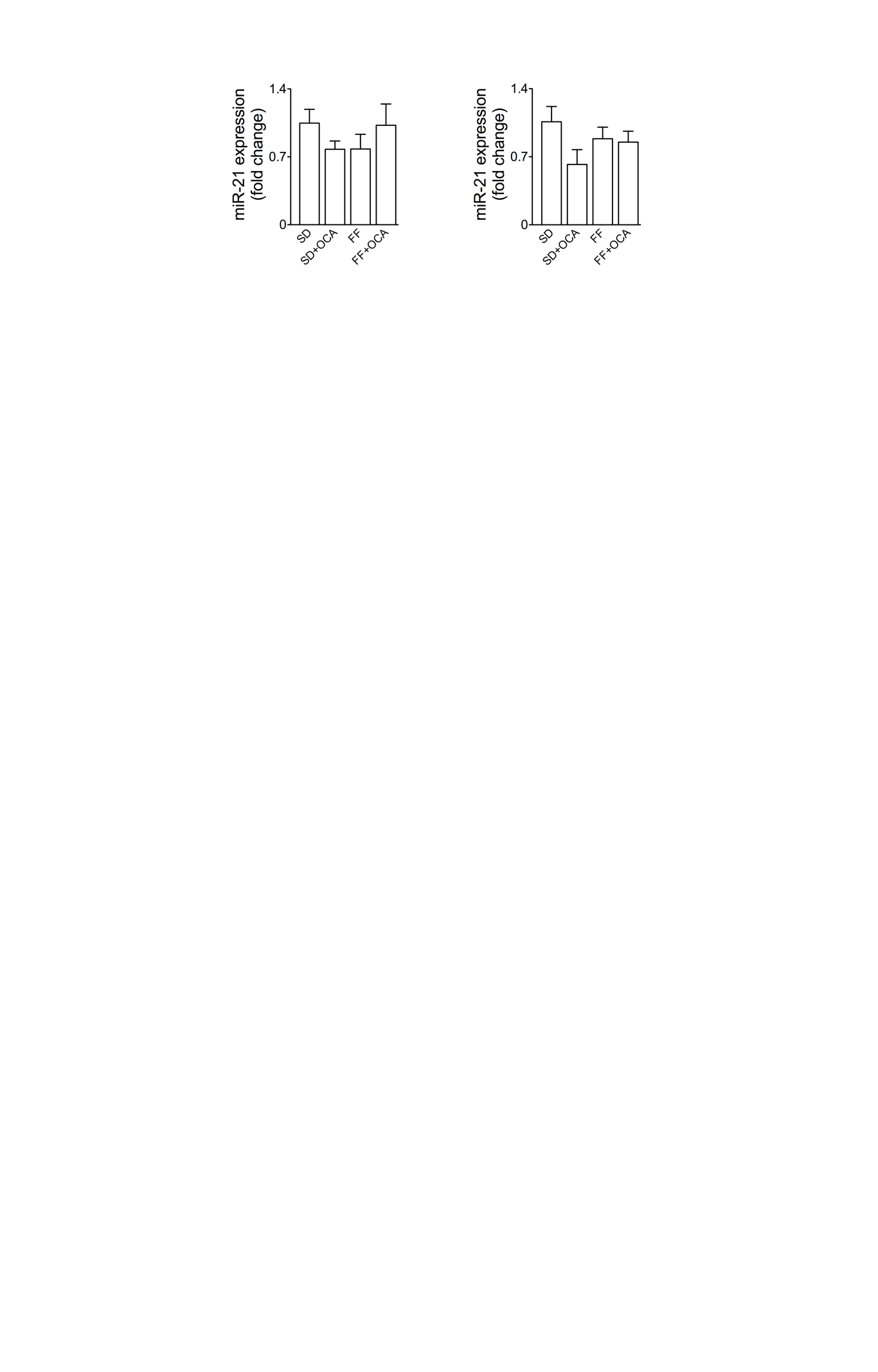


**miR-21 expression is not altered in adipose tissue of mice with NASH.** qRT-PCR analysis of miR-21 expression in visceral (left) and in subcutaneous adipose tissue (right). Results are expressed as mean ± SEM fold change.
